# The sourcil roundness index is a useful measure for quantifying acetabular concavity asphericity

**DOI:** 10.1038/s41598-023-42630-z

**Published:** 2023-09-22

**Authors:** Kenji Kitamura, Masanori Fujii, Goro Motomura, Satoshi Hamai, Shinya Kawahara, Taishi Sato, Ryosuke Yamaguchi, Daisuke Hara, Takeshi Utsunomiya, Yasuhiko Kokubu, Yasuharu Nakashima

**Affiliations:** 1 Department of Orthopaedic Surgery, Graduate School of Medical Sciences, Kyushu University, 3-1-1 Maidashi, Higashi-ku, Fukuoka, 812-8582 Japan; 2 Department of Orthopaedic Surgery, Faculty of Medicine, Saga University, 5-1-1 Nabeshima, Saga, 849-8501 Japan

**Keywords:** Anatomy, Medical research

## Abstract

This study aimed to clarify the clinical utility of the sourcil roundness index (SRI), a novel index for quantifying the asphericity of the acetabular concavity, by determining (1) the difference in the SRI between dysplastic and normal hips and (2) the correlation between the SRI and radiographic parameters of hip dysplasia. We reviewed standing anteroposterior pelvic radiographs of 109 dysplastic and 40 normal hips. The SRI was determined as the ratio of the distance from the medial edge of the sourcil to the most concave point of the acetabular sourcil (A) to the distance from the medial to the lateral edge of the sourcil (B). The formula for SRI is (A/B) × 100–50 (%), with an SRI of 0% indicating a perfectly spherical acetabulum, and higher SRI values indicating a more aspherical shape. The median SRI was greater in patients with hip dysplasia than in normal hips (5.9% vs. − 1.4%; *p* < 0.001). Furthermore, the median SRI was greater in the severe dysplasia subgroup (18.9%) than in the moderate (3.5%) and borderline-to-mild (− 1.3%) dysplasia subgroups (*p* < 0.05). Quantification of acetabular concavity asphericity by the SRI showed that dysplastic hips had a more lateral acetabular concave point than normal hips, and that the severity of hip dysplasia had an effect on the acetabular concavity asphericity.

## Introduction

Hip dysplasia is associated with the worst prognosis for osteoarthritis (OA) progression. It is caused by the concomitant presence of multiple morphological features, including a shallow and steep acetabulum, acetabular undercoverage of the femoral head, and excessive femoral anteversion^1,2^. Although more severe hip dysplasia is likely to progress OA at an earlier age^3,4^, there is often wide variation in OA progression in long-term follow-up, even in patients with a similar degree of hip dysplasia. The factors determining OA progression during the natural course of hip dysplasia are not yet fully understood.

Periacetabular osteotomy (PAO) is considered the gold standard for treating hip dysplasia in skeletally mature patients without advanced OA^5^, which can improve the natural history of patients with hip dysplasia^6^. However, when PAO is performed on congruent hips with an aspherical acetabular concave and femoral head, the congruent relationship between the acetabular concave and femoral head may be disrupted, resulting in poor postoperative joint congruity. This joint incongruity after PAO can impede the improvement in the joint contact pressure, thereby adversely affecting clinical outcomes, which may be attributed to major femoral head deformity^7–10^. In addition, the asphericity of the femoral head has been reported to be influenced by the severity of hip dysplasia^11^. The roundness index of the femoral head (RI) and femoral head sphericity score have been reported as valid radiographic indicators of femoral head deformity^10,12^. Previous studies have also shown that the acetabulum, similar to the femoral head, is aspheric in dysplastic hips compared to normal hips^13,14^, which can lead to joint incongruity. However, no reasonable method exists for assessing the degree of acetabular concavity asphericity without using specialized software.

**Figure 1:**
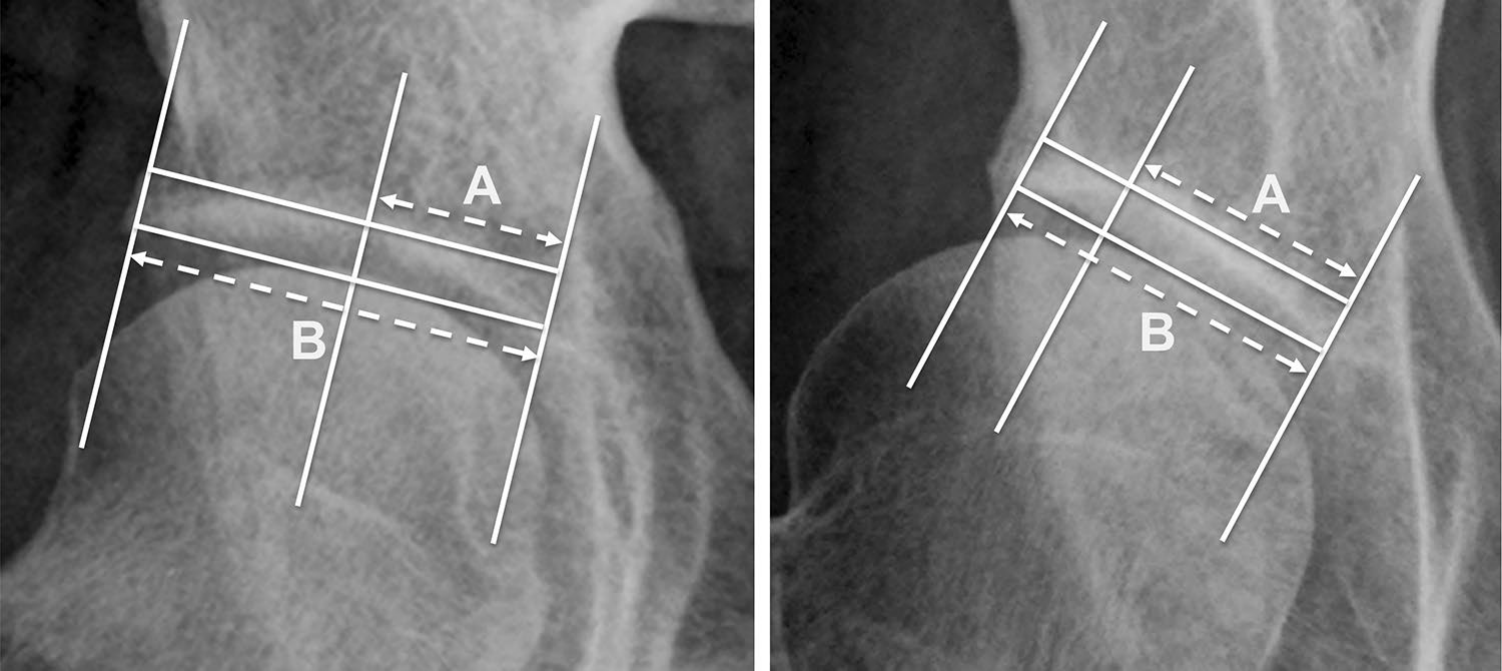
The SRI measurement involves drawing a reference line connecting the lateral and medial edges of the acetabular sourcil. A second line, parallel to the reference line, was drawn through the most concave point of the acetabular sourcil. The SRI is calculated as the ratio of the distance from the medial edge of the acetabular sourcil to the most concave point of the acetabular sourcil (dotted line A) and the distance from the medial edge to the lateral edge of the acetabular sourcil (dotted line B). The formula for the SRI is (A/B) × 100–50 (%). The SRI values range from − 50% if the most concave point was at the medial sourcil edge to + 50% if it was at the lateral sourcil edge. A perfectly spherical acetabulum results in an SRI of 0%, and higher SRI values, whether + or −, correspond to a more aspherical shape. (**a**) The right hip of a 31-year-old women with normal hips has an SRI of − 3.3%, while (**b**) the right hip of an 18-year-old women with moderate dysplasia has an SRI of 25.1%.

Quantitative evaluation of the degree of acetabular concavity asphericity, as well as femoral head deformity, can be useful in predicting joint incongruity after PAO. Therefore, we devised a new radiographic measurement, the “sourcil roundness index (SRI)” (Fig. 1), which quantifies the degree of deformation of the acetabular concavity without requiring specialized software. We sought to determine (1) the intra- and inter-observer reliability of the SRI, (2) the difference in the SRI between dysplastic and normal hips, and (3) the correlation between the SRI and other radiographic measurements of hip dysplasia.

## Results

### Intra- and inter-observer reliability of the SRI

The intra-observer reliabilities of the SRI, evaluated using intraclass correlation coefficients (ICCs), were excellent for both observers (ICC: 0.96 and 0.96), while the inter-observer reliabilities were good or excellent (ICC: 0.88–0.93).

### Comparison of the SRI between dysplastic and normal hips

The SRI was greater in patients with hip dysplasia than in controls (5.9 ± 12.5% vs. − 1.4 ± 8.5%; *p* < 0.001) (Table 1). All radiographic parameters were significantly different between dysplastic and normal hips (Table 1).

**Table 1:** Comparison of radiographic parameters between hip dysplasia and controls.

	hip dysplasia (109 hips)	Controls (40 hips)	*p* value
Lateral center-edge angle (°)^a^	11.1 (− 19 to 24.8)	29.7 (21 to 39.9)	< 0.001
Acetabular roof obliquity (°)^a^	21.4 (5.9 to 34.7)	5.9 (− 10.4 to 14.6)	< 0.001
Sharp angle (°)^a^	48.3 ± 4.1	40.5 ± 4.1	< 0.001
Acetabular head index (%)^a^	64.2 (32.4 to 81.9)	82.6 (73.1 to 93.1)	< 0.001
Anterior wall index^a^	0.276 ± 0.142	0.461 ± 0.131	< 0.001
Posterior wall index^a^	0.909 ± 0.188	1.040 ± 0.138	< 0.001
Crossover sign (+)^b^	20 (18.4)	8 (20.0)	0.82
Posterior wall sign ( +)^b^	70 (64.2)	14 (35.0)	< 0.001
Acetabular depth-to-width ratio^a^	196.5 ± 32.1	277.0 ± 27.2	< 0.001
Sourcil roundness index (%)^a^	5.9 ± 12.5	− 1.4 ± 8.5	0.001
Roundness index of the femoral head (%)^a^	52.3 (47.7 to 59.2)	50.6 (48.6 to 54.7)	< 0.001
Femoro-epiphyseal acetabular roof index (°)^a^	4.3 (− 6.9 to 26.1)	− 9.7 (− 25.1 to − 0.6)	< 0.001

^a^Values are presented as the mean ± standard deviation or the median (range).

^b^Values are presented as the number (%).

### Correlation between the severity of hip dysplasia and SRI

In the hip dysplasia group, the SRI positively correlated with the acetabular roof obliquity, sharp angle, and RI and negatively correlated with the lateral center–edge angle (LCEA), acetabular head index, anterior wall index, and acetabular depth-to-width ratio (ADR) (Table 2). In contrast, in the control group, the SRI correlated negatively with the acetabular roof obliquity and positively with the LCEA, acetabular head index, and ADR (Table 3). The signs of the correlation coefficients in the control group were opposite to those in the hip dysplasia group.

**Table 2:** Correlations between sourcil roundness index and other radiographic parameters in hip dysplasia.

	Correlation coefficient^a^	*p* value
Lateral center-edge angle	− 0.52	< 0.001
Acetabular roof obliquity	0.36	< 0.001
Sharp angle	0.43	< 0.001
Acetabular head index	− 0.51	< 0.001
Anterior wall index	− 0.50	< 0.001
Posterior wall index	− 0.16	0.10
Acetabular depth-to-width ratio	− 0.21	0.03
Roundness index of the femoral head	0.30	0.002
Femoro-epiphyseal acetabular roof index	0.19	0.05

^a^Pearson’s or Spearman’s correlation coefficients.

**Table 3:** Correlations between sourcil roundness index and other radiographic parameters in controls.

	Correlation coefficient^a^	*p* value
Lateral center-edge angle	0.53	< 0.001
Acetabular roof obliquity	− 0.35	0.03
Sharp angle	− 0.26	0.10
Acetabular head index	0.49	0.001
Anterior wall index	0.17	0.29
Posterior wall index	− 0.13	0.43
Acetabular depth-to-width ratio	0.36	0.02
Roundness index of the femoral head	0.19	0.25
Femoro-epiphyseal acetabular roof index	− 0.04	0.83

^a^Pearson’s or Spearman’s correlation coefficients.

The area under the curve (AUC), sensitivity, specificity of the SRI and other radiographic parameters are shown in the supplemental Table S1. The AUC, sensitivity, and specificity of the SRI were 0.67, 98%, and 40%, respectively.

**Figure 2:**
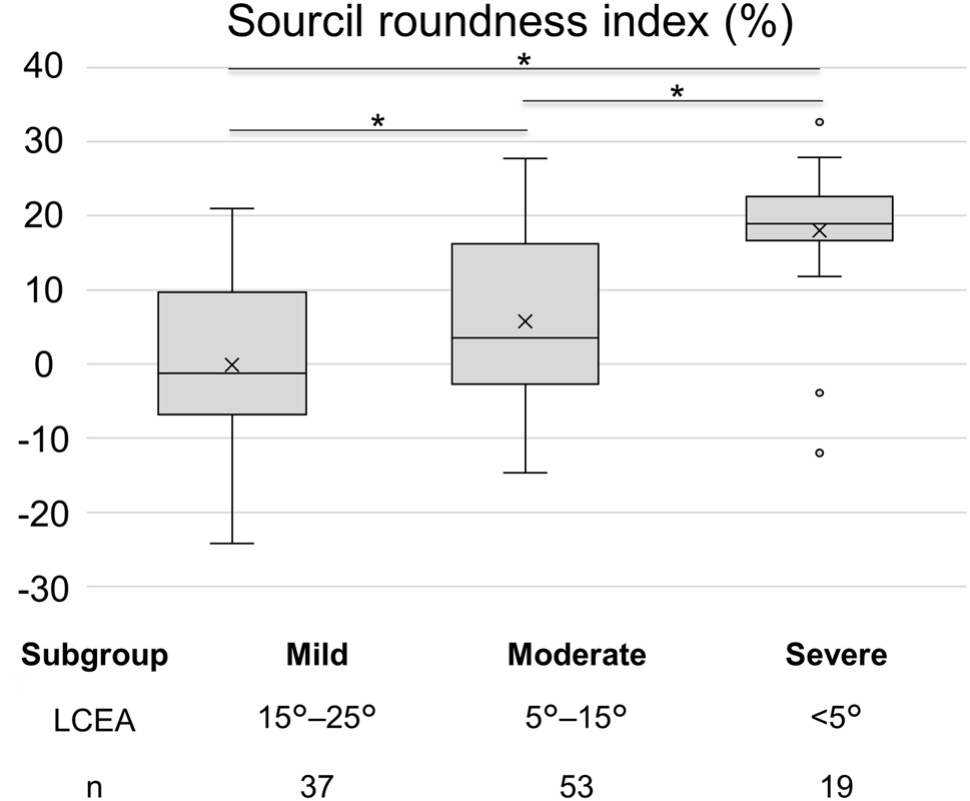
Box-and-whisker diagrams showing the distribution of the sourcil roundness index (SRI) according to the severity of hip dysplasia. Significant differences (*p < 0.05) were found between all subgroups, i.e., the more severe the degree of hip dysplasia, the greater the SRI. LCEA; Lateral center–edge angle.

Hip dysplasia was severe (LCEA: < 5°) in 19 hips, moderate (LCEA: 5°–15°) in 53 hips, and borderline-to-mild (LCEA: 15°–25°) in 37 hips. The median SRI was greater in the severe subgroup than in the moderate subgroup (18.9% [− 12.0–33.4] vs. 3.5% [− 14.7–27.8]; *p* < 0.001), whereas it was greater in the moderate subgroup than in the borderline-to-mild subgroup (3.5% [− 14.7–27.8] vs. − 1.3% [− 24.2–21.0]; *p* = 0.04; Fig. 2).

Of the 109 dysplastic hips in 56 patients, 76 hips were symptomatic and 33 hips were asymptomatic. There was no difference in the SRI between symptomatic and asymptomatic hips (3.6% [− 24.2–33.4] vs. 3.6% [− 15.2–22.4]; *p* = 0.31).

## Discussion

When performing PAO, joint congruency is an important predictor of postoperative outcomes ^9,15^. However, in cases where PAO is performed on hips with an aspheric acetabular concave and femoral head, the congruent relationship between the acetabular concave and femoral head may be disrupted, resulting in poor postoperative joint congruity. Morita et al.^15^ developed an original software to define the “center gap”, which measures the distance between the acetabular rotation center and the femoral head center, and reported it to be an independent risk factor for OA progression. Femoral head deformity is a well-documented cause of joint incongruity between the femoral head and the reoriented acetabulum after PAO^7,9,10,16^. Okano et al.^10^ showed that in patients with a preoperative RI > 64%, OA progressed within 10 years after PAO, even in patients with early-stage OA. Previous studies have also shown that the acetabulum, similar to the femoral head, is aspheric in dysplastic hips^13,14^, which can lead to joint incongruity. Irie et al.^14^ demonstrated alteration in three-dimensional (3D) regional acetabular curvature in dysplastic hips using original software; however, there is currently no viable method for assessing the degree of acetabular concavity asphericity without specialized software. Therefore, we have developed a new radiographic measurement, the SRI, to quantify the degree of the acetabular concavity asphericity.

The results of this study showed that the ICCs for the intra- and inter-observer reliability of the SRI were good or excellent, indicating its high reproducibility. Our findings demonstrate that the SRI significantly differed between dysplastic and normal hips: The SRI was greater in patients with dysplastic hips than in those with normal hips. This means that the most concave point of the acetabular sourcil is more lateralized in hip dysplasia, similar to the most protruding point of the femoral head^11^. We speculate that these deformities are influenced by the interrelationship of the femoral head and acetabulum during growth. The acetabulum requires a spherical femoral head as a template for spherical growth, and vice versa, maintaining an interdependent relationship in the developmental environment of a congruent hip^17,18^. However, deficient acetabular coverage in hip dysplasia can cause asymmetric epiphyseal growth, resulting in an oval-shaped femoral head^16^. Consequently, the concave shape of the acetabular sourcil is expected to become aspherical. The correlation between the RI and SRI in this study may support the interdependent relationship in the growth process.

A previous study reported that the RI, an indicator of femoral head deformity, correlated with the LCEA, acetabular roof obliquity, sharp angle, and acetabular head index in hip dysplasia^11^. In the present study, the SRI correlated with most radiographic parameters of hip dysplasia, except the posterior wall index and the femoro-epiphyseal acetabular roof (FEAR) index. While we acknowledge that the correlations between the SRI and conventional dysplasia parameters were not particularly strong, it is important to note that the SRI offers a unique perspective on acetabular concavity asphericity that is not captured by these traditional parameters. Contrary to hip dysplasia, a positive correlation was found between the SRI and the indicators of the lateral coverage of the acetabulum, i.e., LCEA and acetabular head index in the control group. This implies that in hip dysplasia, the smaller the lateral acetabular coverage, the greater the SRI, while in normal hips, the larger the acetabular lateral coverage, the greater the SRI. The reason for the inverse correlation of these parameters between dysplastic and control hips is unknown. Future studies evaluating the SRI in various hip diseases, including femoroacetabular impingement (FAI), may help us to better understand the role of the acetabular concavity asphericity in the pathogenesis of hip diseases.

Although the severity of hip dysplasia affects the deformity of the femoral head^11^, the present study shows that the severity of hip dysplasia also affects the acetabular concavity asphericity. Thus, patients with severe hip dysplasia tend to present with aspherical but congruent hip joints. When PAO is performed on congruent hips with aspherical acetabular concave and femoral head, the interrelationship between originally congruent acetabular concave and femoral head may be disrupted, leading to postoperative OA progression due to incongruity and acetabular undercoverage. The SRI may be useful in predicting such postoperative incongruity and the natural course of joint prognosis after PAO, but further studies are needed to clarify its clinical significance.

There are several limitations to this study. First, the selected radiological parameters, including the SRI, were based solely on a two-dimensional (2D) assessment. However, hip joint congruity is predicated on a 3D configuration, and the usefulness of the SRI may depend on whether it is a reliable surrogate for the 3D configuration. Furthermore, anteroposterior (AP) pelvic radiographs do not accurately depict the anteroposterior axis, and magnification error may affect distances in the medial-to-lateral and superior-to-inferior axes^19^. However, AP pelvic radiographs are the most versatile and convenient tool for pre- and postoperative assessment, and the SRI can be measured without specialized software, thus rendering it universally available. Future studies should verify the correlation of the SRI with CT-based assessment of 3D acetabular asphericity and joint incongruity. Second, the diagnostic performance of the SRI was not superior to that of other radiographic parameters, and the SRI did not exhibit strong correlations with these parameters. Therefore, the diagnostic potential of the SRI compared with existing indicators remains uncertain. However, the SRI provides a unique perspective on acetabular concavity asphericity that is not captured by traditional parameters, and it is currently the only method to quantify the acetabular concavity asphericity without relying on specialized software. Future research is needed to further explore the potential clinical implications of the SRI, such as its utility in preoperative planning for PAO and its role in predicting postoperative joint prognosis. Third, there is a potential for error in the radiographic measurement of the SRI related to pelvic rotation. Ideally, the pelvis should be in a neutral position to allow accurate measurement of the SRI. Extreme internal or external rotation of the pelvis can lead to morphological changes in the acetabular concave, resulting in underestimation or overestimation of the SRI. However, the present study did not include cases with extreme pelvic rotation. Lastly, the sample size was relatively small, especially in the severe subgroup, which may have limited our ability to detect potential differences. However, post hoc power analysis showed adequate power (> 99% power at *p* = 0.05) to detect a difference in SRI between the three subgroups (Fig. 2). Moreover, this study included only young patients who had undergone PAO, with symptomatic hip dysplasia on at least either side and without advanced OA, which may be one of the reasons why the SRI could not be shown to be related to symptoms or OA progression. Further studies are needed to include a variety of subjects with hip dysplasia, including asymptomatic individuals of all generations.

In conclusion, this study suggests that the SRI is a convenient index for evaluating acetabular concavity on plain radiographs without specialized software. This study reveals that the acetabular morphology of dysplastic hips is characterized by a more lateralized concave point of acetabular sourcil than that of normal hips. In addition, the more severe the hip dysplasia, the greater the acetabular concavity asphericity. These findings may be useful in preoperative planning of PAO and predicting the natural course of joint prognosis after PAO. Future clinical studies on the biomechanical analysis of dysplastic hips and the natural history of joint prognosis after PAO are needed to validate the clinical significance of the SRI.

## Methods

### Ethical committee approval

The authors certify that their institutions approved the human protocol for this investigation and that all investigation was conducted in conformity with ethical principles of research. Ethical approval for this study was obtained from the Graduate School of Medical Sciences, Kyushu University (approval number 30-137). Informed consent was obtained from all participants included in the study, and all participants were informed of the radiation exposure required.

### Patients

Preoperative standing AP pelvic radiographs of 72 patients with symptomatic hip dysplasia (LCEA on supine AP pelvic radiographs < 25°) who underwent unilateral PAO at our institution between November 2018 and April 2021 were retrospectively reviewed. Among these patients, 16 were excluded for the following reasons: four had no available radiographs, seven had advanced osteoarthritis on either joint, and five had a history of surgery on either joint. Of the remaining 112 hips in 56 patients, three hips were excluded because the LCEA was within the normal range (LCEA ≥ 25°). Thus, 109 hips from 56 patients (3 males and 53 females; mean age, 38.3 years) were finally eligible for this study (Table 4). There were no hips with major femoral head deformities and obvious cam lesions on the Lauenstein view. The severity of hip dysplasia was classified into three subgroups based on LCEA measurements as follows: severe (LCEA < 5°), LCEA: 5°–15°, moderate (5° ≤ LCEA < 15°), and borderline-to-mild (15° ≤ LCEA < 25°)^20,21^.

**Table 4:** Demographic and radiographic parameters in hip dysplasia and control groups.

Parameters	Hip dysplasia n = 56 patients (109 hips)	Controls n = 22 patients (40 hips)	*p* value
Age (years)^a^	38.3 ± 10.9	35.4 ± 5.9	0.06
Sex (male/female)^b^	3 (5.4)/53 (94.6)	3 (13.6)/19 (86.4)	0.24
Body mass index (kg/m^2^)^a^	22.6 ± 4.1	21.3 ± 2.7	0.32
Height (cm)^a^	158.1 ± 5.9	160.5 ± 7.8	0.46
Weight (kg)^a^	56.5 ± 10.9	54.6 ± 8.6	0.42
Laterality (right/left hip)^b^	54 (49.5)/55 (50.5)	21 (52.5)/19 (47.5)	0.75
Bilateral hip dysplasia^b^	53 (94.6)	0 (0)	< 0.001
Bilateral hip symptom^b^	20 (35.7)	0 (0)	< 0.001
Tönnis classification system (grade 0/1)^b^	79 (72.5)/30 (27.5)	40 (100)/0 (0)	< 0.001
Lateral center-edge angle (°)^a^	11.2 ± 7.9	31.1 ± 3.3	< 0.001

^a^Values are presented as the mean ± standard deviation.

^b^Values are presented as the number (%).

For the control group, we reviewed the data of nine healthy male and 33 healthy female volunteers included in prior studies^22,23^. Based on medical interviews, AP pelvic radiographs, and pelvic computed tomography scans, none of the patients had a history of disease or articular symptoms in their hips. Seventeen hips with frank or borderline hip dysplasia (LCEA: < 25°), one hip with FAI (LCEA: > 40°), and 26 hips in 13 patients without appropriate images were excluded. Thus, 40 hips in 22 individuals (three males and 19 females) were included in the control group (Table 4).

### Radiographic parameters

Radiographic parameters, including the SRI, were assessed using preoperative standing AP pelvic radiographs taken prior to PAO. All parameters were measured using 2D template software (Kyocera Medical Corporation, Osaka, Japan). The following conventional radiographic parameters were used: LCEA, acetabular roof obliquity, sharp angle, acetabular head index, anterior and posterior wall index, Crossover sign, posterior wall sign, ADR, FEAR index, and RI^10,24–27^. Our previous study demonstrated that the intra- and inter-observer reliabilities of these parameters, assessed using kappa values and ICCs, were good or excellent^28^.

### Measurement of the SRI

The SRI measurement involves drawing a reference line connecting the lateral and medial edges of the acetabular sourcil. A second line, parallel to the reference line, was drawn through the most concave point of the acetabular sourcil. The SRI is calculated as the ratio of the distance from the medial edge of the acetabular sourcil to the most concave point of the acetabular sourcil (Fig. 1, dotted line A) and the distance from the medial edge to the lateral edge of the acetabular sourcil (Fig. 1, dotted line B). The formula for the SRI is (A/B) × 100–50 (%). The SRI values range from − 50% if the most concave point was at the medial sourcil edge to + 50% if it was at the lateral sourcil edge. A perfectly spherical acetabulum results in an SRI of 0%, and higher SRI values, whether + or −, correspond to a more aspherical shape.

### Statistics

All statistical analyses were performed using JMP^®^ version 16.0 (SAS Institute). A t-test or Wilcoxon rank-sum test was used to compare continuous parameters between the hip dysplasia and control groups after confirming normal distribution and homoscedasticity using the Shapiro–Wilk W and F tests. The chi-square test was used to compare categorical parameters between the two groups. The Tukey–Kramer honestly significant difference test or the Steel–Dwass test was used for multiple comparisons, as appropriate. Differences were considered significant when the *p*-value was < 0.05. Correlations between two continuous parameters were evaluated using Pearson’s or Spearman’s correlation coefficient, as appropriate. Receiver operating characteristic curves were used to identify the diagnostic performance of the SRI and other radiographic parameters to determine the presence of hip dysplasia. AUC, sensitivity, and specificity were determined using the Youden index.

Two board-certified orthopedic surgeons (KK and MF) measured the SRI. To test intra-observer reliability, we repeated the measurements in a blind test on randomly selected hips > 2 weeks later. To test inter-observer reliability, two independent observers (KK and MF) performed measurements on 20 randomly selected hips in a blind test, in which a simple sampling technique through a computer-generated random number was used. The intra- and inter-observer reliabilities were assessed using ICCs, which were interpreted as follows: ICC: < 0.30, negligible; ICC: 0.31–0.50, weak; ICC: 0.51–0.70, moderate; ICC: 0.71–0.90, good; and ICC: > 0.90, excellent.

### Supplementary Information

Supplementary Table S1.

## Data Availability

Data is available on reasonable request via contacting the corresponding authors.
