# Supplementary material for: The sourcil roundness index is a useful measure for quantifying acetabular concavity asphericity
Source: Sci Rep. 2023 Sep 22;13:15851. doi: 10.1038/s41598-023-42630-z (PMC10516987; doi:10.1038/s41598-023-42630-z)
Supplement: Supplementary file 1 — Supplementary Table S1. [file 41598_2023_42630_MOESM1_ESM.docx]

**Supplemental Table.** Comparison of the validity of the sourcil roundness index and other radiographic parameters for detecting hip dysplasia

|  | Area under the curve | Sensitivity (%) | Specificity (%) |
| --- | --- | --- | --- |
| Lateral center-edge angle | 0.99 | 100 | 95 |
| Acetabular roof obliquity | 0.99 | 98 | 94 |
| Sharp angle | 0.91 | 93 | 81 |
| Acetabular head index | 0.99 | 98 | 97 |
| Anterior wall index | 0.83 | 85 | 69 |
| Posterior wall index | 0.69 | 98 | 34 |
| Crossover sign | 0.51 | 20 | 82 |
| Posterior wall sign | 0.65 | 65 | 64 |
| Acetabular depth-to-width ratio | 0.95 | 93 | 90 |
| Sourcil roundness index | 0.67 | 98 | 40 |
| Roundness index of the femoral head | 0.74 | 78 | 67 |
| Femoro-epiphyseal acetabular roof index | 0.98 | 93 | 94 |
